# IRF9对伴PML-RARα急性早幼粒细胞白血病细胞生物学功能的影响

**DOI:** 10.3760/cma.j.issn.0253-2727.2022.05.004

**Published:** 2022-05

**Authors:** 雪 杨, 海燕 邢, 克晶 唐, 征 田, 青 饶, 敏 王, 建祥 王

**Affiliations:** 中国医学科学院血液病医院（中国医学科学院血液学研究所），实验血液学国家重点实验室，国家血液系统疾病临床医学研究中心，细胞生态海河实验室，天津市血液病细胞治疗研究重点实验室，天津 300020 杨雪现在复旦大学附属中山医院血液科，上海 200030 State Key Laboratory of Experimental Hematology, National Clinical Research Center for Blood Diseases, Haihe Laboratory of Cell Ecosystem, Tianjin Key Laboratory of Cell Therapy for Blood Diseases, Institute of Hematology & Blood Diseases Hospital, Chinese Academy of Medical Sciences & Peking Union Medical College, Tianjin 300020, China Yang Xue is working at the Department of Hematology, Zhongshan Hospital, Fudan University, Shanghai 200030, China

**Keywords:** 白血病，早幼粒细胞，急性, 干扰素调节因子, 生物学功能, Leukemia, promyelocytic, acute, Interferon regulatory factor, Biological function

## Abstract

**目的:**

研究急性早幼粒细胞白血病（APL）中干扰素调节因子9（IRF9）的表达特征、预后意义和生物学功能，探索IRF9作为潜在治疗靶点的临床转化意义。

**方法:**

挖掘TCGA公共数据分析IRF9在APL中的表达水平及其表达高低对患者生存的影响；利用Dox诱导的慢病毒载体系统构建可诱导表达PML-RARα（PR）融合基因的U937细胞系，诱导PR融合基因早期表达并加入全反式维甲酸（ATRA）检测IRF9表达变化；构建可诱导表达IRF9的NB4细胞系，进行细胞分化和集落形成实验，初步探讨IRF9诱导表达对NB4白血病细胞生物学功能的影响。

**结果:**

①TCGA数据库分析显示，在各种类型的AML中，IRF9在APL中表达水平最低且与预后不良相关。②成功构建可诱导表达PR融合基因的U937细胞系；PR表达引起IRF9蛋白水平下调，在NB4细胞系中IRF9表达缺失，ATRA处理后可表达上调。③成功构建可诱导表达IRF9的NB4细胞系，IRF9诱导表达促进NB4细胞的分化，且与较低剂量ATRA有协同促分化作用；IRF9诱导表达显著抑制了NB4细胞的集落形成能力。

**结论:**

IRF9在APL中低表达且与预后不良相关，提示存在特异性调控机制；上调IRF9表达可发挥抗白血病效应，为其成为临床潜在治疗靶点奠定生物学基础。

## Abstract (en)

**Objective:**

To investigate the prognostic significance of interferon regulatory factor 9（IRF9）expression and identify its role as a potential therapeutic target in acute promyelocytic leukemia（APL）.

**Methods:**

The gene expression profile and survival data applied in the bioinformatic analysis were obtained from The Cancer Genome Atlas and Beat acute myeloid leukemia（AML）cohorts. A dox-induced lentiviral system was used to induce the expression of PML-RARα（PR）in U937 cells, and the expression level of IRF9 in U937 cells treated with or without ATRA was examined. We then induced the expression of IRF9 in NB4, a promyelocytic leukemia cell line. In vitro studies focused on leukemic phenotypes triggered by IRF9 expression.

**Results:**

①Bioinformatic analysis of the public database demonstrated the lowest expression of IRF9 in APL among all subtypes of AML, with lower expression associated with worse prognosis. ②We successfully established a PR-expression-inducible U937 cell line and found that IRF9 was downregulated by the PR fusion gene in APL, with undetectable expression in NB4 promyelocytic cells. ③An IRF9-inducible NB4 cell line was successfully established. The inducible expression of IRF9 promoted the differentiation of NB4 cells and had a synergistic effect with lower doses of ATRA. In addition, the inducible expression of IRF9 significantly reduced the colony formation capacity of NB4 cells.

**Conclusion:**

In this study, we found that the inducible expression of PR downregulates IRF9 and can be reversed by ATRA, suggesting a specific regulatory relationship between IRF9 and the PR fusion gene. The induction of IRF9 expression in NB4 cells can promote cell differentiation as well as reduce the colony forming ability of leukemia cells, implying an anti-leukemia effect for IRF9, which lays a biological foundation for IRF9 as a potential target for the treatment of APL.

急性早幼粒细胞白血病（APL）是急性髓系白血病（AML）中的特殊类型，98％的患者存在PML-RARα（PR）融合基因[1]。全反式维甲酸（ATRA）联合亚砷酸（ATO）治疗使多数APL患者得以治愈，但仍有少数患者耐药或复发[2]。因此，探索APL的分子机制和新的治疗靶点具有实际临床意义。干扰素调节因子（IRF）是一类调控干扰素（IFN）和干扰素刺激基因（ISG）表达的转录因子。当前关于IRF的研究多集中于其对固有免疫和适应性免疫反应的调节作用，IRF与肿瘤发生直接相关的研究则较少。较之于其他8种IRF，干扰素调节因子9（IRF9）因研究较少被称为“被遗忘的干扰素调节因子”[3]，在血液肿瘤中缺乏报道。本研究中，我们利用公共数据库分析IRF9在APL中的表达和预后意义，利用Tet-on可诱导表达系统构建表达IRF9的白血病细胞系，并通过体外功能实验初步探讨其抗白血病作用。

## 材料与方法

一、主要材料与试剂

RPMI 1640培养基、DMEM培养基、胎牛血清（FBS）购于美国GIBCO公司；MethoCult™ H4230购于加拿大 Stemcell technologies 公司；无内毒素质粒小提试剂盒、胶回收试剂盒、DNA片段回收试剂盒、Prime Starmax PCR Master mix购于北京TaKaRa生物技术公司；无内毒素质粒中提试剂盒购于北京天根生化科技有限公司；T4连接酶和限制性内切酶购于英国NEB公司；RNA提取试剂盒、逆转录试剂盒、qPCR试剂盒购于北京全式金生物技术有限公司；pLVX-Tight-Puro质粒购于北京TaKaRa生物技术公司，pLVX-Tet-On Advanced-GFP质粒为本实验室改造构建，psPAX2质粒和pMD.2G质粒购于美国SBI公司；IRF9兔单克隆抗体购于美国CST公司；强力霉素（Doxycycline, Dox）、嘌呤霉素（Puromycin）、ATRA、Flag单克隆抗体、β-actin小鼠单克隆抗体购于德国Sigma Aldrich公司；Annexin Ⅴ-Alexa Fluor® 647、Annexin Ⅴ Binding Buffer、PE标记的抗人CD11b抗体、PE Mouse IgG1κ同型对照抗体购于美国Biolegend公司；碘化丙啶（PI）、细胞周期检测试剂盒购于上海碧云天生物技术有限公司。

二、数据来源及生物信息学分析

TCGA表达谱数据以及患者生存数据均来自cBioPortal数据库（http://www.cbioportal.org/）。将原始的count表达谱数据通过标准化（Normalization）转化为CPM（Count Per Million）值，下游表达量的比较及生存分析均基于CPM值进行。利用Kruskal-Wallis检验比较不同FAB分型患者表达谱数据中IRF9的CPM值，利用Wilcoxon检验比较APL患者及非APL患者表达谱数据中IRF9的CPM值，依据*α*＝0.05判断差异是否有统计学意义。以所有APL患者IRF9表达值的中位数作为阈值，将APL患者分为高表达IRF9组和低表达IRF9组，通过Log-rank检验比较两组之间的生存差异。

三、细胞培养

人白血病细胞系U937细胞、可诱导表达PR融合基因的U937细胞系（U937-PR）、人APL细胞系NB4、可诱导表达IRF9的NB4细胞系（NB4-IRF9）及人白血病细胞系Kasumi-1细胞培养于含10％ FBS的RPMI 1640培养基中；人胚肾细胞系HEK-293T培养于含10％ FBS的DMEM培养基中。

四、可诱导表达白血病细胞系的构建

以实验室前期构建的MSCV-PML-RARα-Flag-IRES-GFP质粒为模板设计带NotⅠ和EcoRⅠ酶切位点的引物，扩增PML-RARα-Flag片段，正向引物：5′-AAGGAAAAAAGCGGCCGCATGGAGCCTGCACCCG-3′；反向引物：5′-CCGAATTCTCACTTATCGTCGTCATCCTTGTAATCGTTAA-3′。PCR产物纯化后酶切连接pLVX-Tight-Puro载体，转化后提质粒送测序鉴定pLVX-Tight-Puro-PML-RARα-Flag（pLVX-PR）构建成功。将调控质粒pLVX-Tet-on-Advanced-GFP（rtTA-GFP）和表达质粒pLVX-PR分别转染293T细胞进行病毒包装，获取新鲜病毒浓缩液后进行后续的感染实验。首先将rtTA-GFP病毒感染U937细胞，48 h后流式细胞术分选GFP阳性细胞获得表达调控质粒的稳定克隆（U937-rtTA）。用表达目的序列的pLVX-PR病毒感染U937-rtTA，感染48 h后使用2.5 µg/ml的嘌呤霉素（puro）进行药物筛选，随后在细胞培养体系中加入500 ng/ml Dox诱导PR融合蛋白表达，成功构建U937-PR。以Kasumi-1细胞系为模板扩增IRF9 CDS区，同上步骤构建NB4-IRF9。

五、IRF9表达对NB4细胞生物学功能的影响

1. 细胞分化：将NB4-IRF9细胞按2×10^5^/ml的密度铺板，分别于24、48和72 h收集细胞检测表面分化抗原。140×*g*离心5 min，弃上清。100 ml PBS重悬细胞，每1×10^6^细胞加入2 ml PE-CD11b抗体，对照组加入2 ml PE同型对照抗体，室温避光孵育30 min。加入1 ml PBS洗去残留的抗体，离心3 min，弃上清。加入200 µl PBS重悬细胞，流式细胞仪检测CD11b阳性细胞的比例，细胞甩片及瑞式染色，观察分化细胞形态。实验重复3次。

2.集落形成实验：将NB4细胞重悬于RPMI 1640培养基，调整细胞密度至4×10^3^/ml。各组取200 µl细胞悬液分别放入1.5 ml EP管中，每管中加入20 µl双抗。将细胞悬液加入2 ml甲基纤维素培养基H4230，vortex混匀。混匀后的细胞与H4230吸入到注射器中，以每孔500 µl（200个细胞/孔）加入到24孔板中，每组3个复孔，避免气泡产生。24孔板中的周围孔中加入PBS，37 °C、5％ CO_2_孵箱培养7 d后计数，高内涵成像拍摄集落图。实验重复3次。

六、统计学处理

采用R语言和Graphpad Prism 8进行作图及统计分析，计量资料以均值±标准差表示，组间比较采用*t*检验。使用Image J进行蛋白条带灰度值分析并计算相对表达量（RI）。*P*<0.05为差异有统计学意义。

## 结果

1. IRF9在APL中的表达及预后意义：查询TCGA公共数据库中白血病患者表达谱数据，结果显示在不同FAB分型的AML中，IRF9在APL（M_3_）中表达最低（图1A）。APL患者IRF9表达水平显著低于非APL AML患者，差异具有统计学意义（4.955±0.536对5.30±0.662，*P*＝0.021）。生存分析显示，APL患者中IRF9高表达组与低表达组总生存（OS）差异无统计学意义（*P*>0.05），但IRF9高表达的患者OS时间趋于更长，IRF9低表达的患者则趋于预后不良（图1B）。

**图1:**
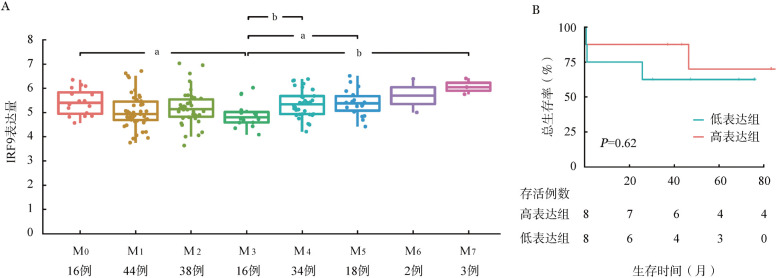
TCGA数据分析IRF9在急性髓系白血病中的表达（A）及其在急性早幼粒细胞白血病中与预后的关系（B） 与M_3_组比较，^a^ *P*<0.05，^b^ *P*<0.01

2. 可诱导表达PR白血病细胞系的构建及蛋白表达：利用Dox诱导的慢病毒载体系统构建了可诱导表达PR的U937细胞系（U937-PR）（图2A），经梯度预实验后选择可稳定表达PR融合蛋白的浓度及时间，即500 ng/ml Dox诱导48 h（图2B）。PR诱导表达直接引起IRF9蛋白水平下调，在此基础上使用ATRA处理24 h，PR下调的IRF9可被恢复（图2C）。在NB4细胞系中IRF9本底表达缺失。加入ATRA和ATO处理后，ATRA可逐渐上调IRF9的表达水平，但该作用未见于ATO处理的细胞（图2D）。

**图2:**
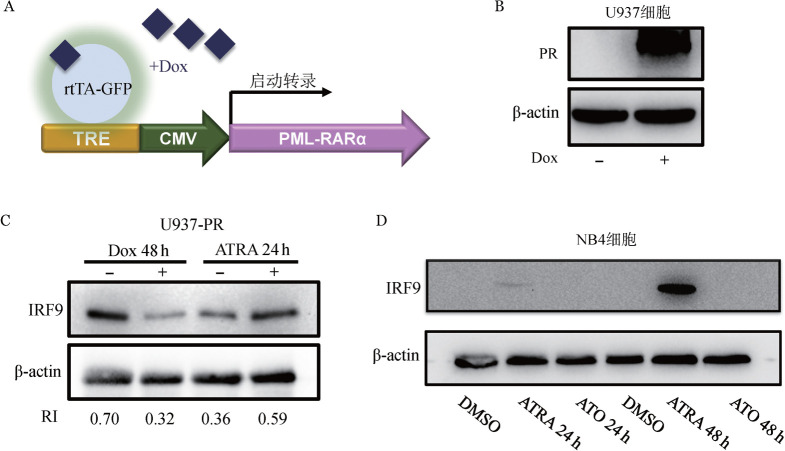
可诱导表达PML-RARα（PR）细胞系的构建及药物处理后IRF9蛋白表达 A：可诱导表达细胞系U937-PR载体构建示意图；B：Western blot验证PR融合蛋白过表达；C：IRF9在U937-PR细胞中表达下调且被全反式维甲酸（ATRA）恢复表达水平；D：IRF9在NB4细胞中的表达及ATRA和亚砷酸（ATO）对其的影响。Dox：强力霉素；RI：IRF9蛋白相对表达量

3. 可诱导表达IRF9白血病细胞系的建立：为了研究IRF9在APL中的功能表型，进一步利用Dox诱导的慢病毒载体构建了可诱导表达IRF9的NB4细胞系（NB4-IRF9）（图3A）。在NB4培养基中加入Dox 1 µg/ml可使IRF9在NB4细胞中稳定表达72 h（图3B）。

**图3:**
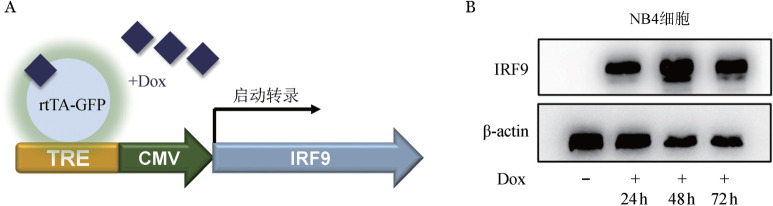
可诱导表达细胞系NB4-IRF9载体构建示意图（A）及Western blot验证IRF9过表达（B） Dox：强力霉素

4. IRF9表达对NB4细胞分化功能的影响：在NB4细胞中加入Dox诱导IRF9的表达，并分别在24、48和72 h收集细胞用流式细胞术检测髓系分化标志CD11b的表达水平。结果显示，诱导表达IRF9可促进NB4细胞分化。IRF9诱导表达48、72 h后CD11b表达水平显著高于对照组（*P*值分别为0.002和0.003）（图4A）。IRF9诱导表达与较低剂量ATRA（10 nmol/L和100 nmol/L）有协同促分化作用，其中IRF9联合10 nmol/L ATRA作用24、48、72 h组的CD11b表达水平较对照组差异有统计学意义（*P*值分别为0.041、0.006、0.008）（图4B）；IRF9联合100 nmol/L ATRA作用48、72 h组的CD11b表达水平较对照组差异有统计学意义（*P*值分别为0.030和0.001）（图4C）。由于NB4细胞对ATRA的促分化作用具有高度敏感性，较高剂量的1 µmol/L ATRA在48 h即可诱导NB4细胞终末分化，因此过表达IRF9并未与1 µmol/L ATRA产生明显的分化协同作用（图4D）。72 h对应的典型形态学染色如图5所示。

**图4:**
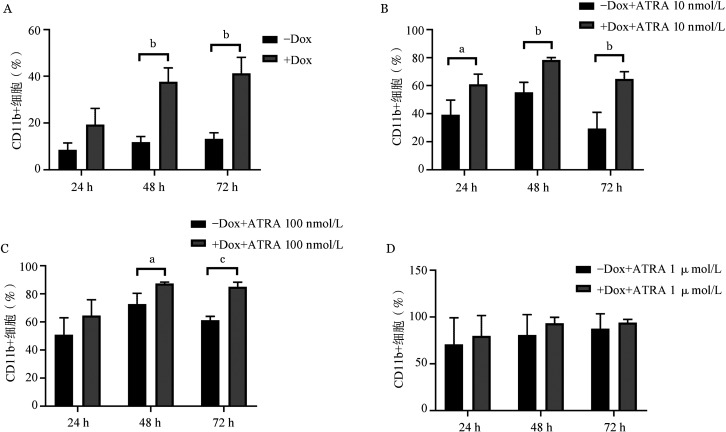
IRF9诱导表达（A）及协同10 nmol/L（B）、100 nmol/L（C）和1 µmol/L（D）全反式维甲酸（ATRA）对NB4细胞分化的影响（实验重复3次） 两组比较，^a^ *P*<0.05，^b^ *P*<0.01，^c^ *P*<0.001

**图5:**
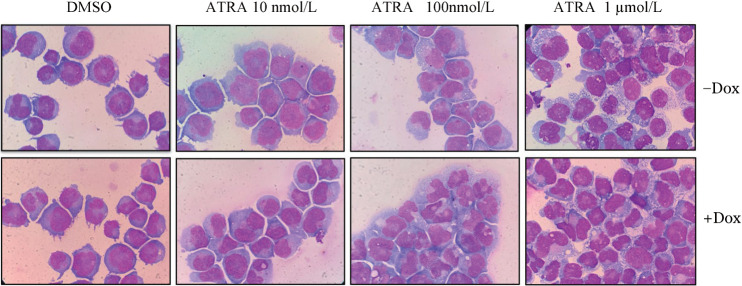
IRF9在NB4细胞中诱导表达及药物处理72 h典型形态图 ATRA：全反式维甲酸

5. IRF9表达对NB4细胞集落形成能力的影响：在NB4细胞中诱导IRF9表达，培养24 h后将诱导前后的NB4细胞在H4230半固体培养基中进行集落形成实验。结果显示IRF9过表达后，NB4细胞的集落形成数量和大小均被显著抑制（55.00±7.81对 20.00±4.58，*P*＝0.003）。实验组（+Dox）和对照组（−Dox）高内涵成像和荧光显微镜的典型局部和全景集落图如图6所示。

**图6:**
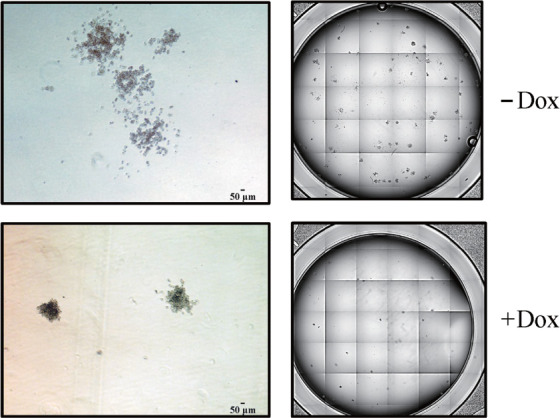
IRF9表达对NB4细胞集落形成能力的影响

## 讨论

IRF是IFN通路上一类重要的转录因子，在驱动抗病毒反应和调节免疫细胞分化等多方面参与免疫反应的调控[4]。近年来的研究指出IRF对细胞生物学功能的影响不仅仅局限于抗病毒免疫反应，IRF亦可调节细胞增殖、凋亡和肿瘤发生[5]。IRF9通常与STAT1/STAT2形成ISGF3复合体发挥作用[6]。在本研究中，我们发现在各种类型的AML中，IRF9在APL中表达水平最低。IRF9可被PR融合蛋白诱导下调，在NB4细胞中表达缺失；诱导IRF9表达可促进NB4细胞分化并抑制白血病细胞的集落形成能力，提示IRF9表达具有抗白血病效应。有研究报道和正常人相比，IRF9在AML中表达下调。在OCI-AML-2和OCI-AML-3细胞中敲降IRF9促进细胞增殖和集落形成，过表达则可逆转白血病表型[7]。作为融合基因驱动的白血病，APL是AML中的特殊类型。IRF9在APL中的差异表达与功能表型提示PR融合基因与IRF9之间可能存在独特的调控机制。

目前已有多项研究揭示了IRF8作为肿瘤抑制基因参与APL发生发展的作用机制。一项研究显示IRF8敲除鼠可呈现骨髓增殖性肿瘤（MPN）表型，而在MRP8-PML/RARα转基因小鼠中敲除IRF8则进一步加速了APL的发生，同时缩短了小鼠生存[8]，另一项研究在APL小鼠模型中过表达IRF8则显示小鼠的生存显著延长[9]。2010年的一项关键研究对NB4细胞系和APL患者原代细胞进行染色质免疫共沉淀测序（ChIP-seq），测序结果显示IRF1和IRF8均为PR融合基因结合的靶基因[10]。然而在PR结合的基因数据中未见IRF9，提示IRF9可能并非PR直接调控的靶基因。早期文献报道ATRA处理NB4细胞时IRF1的表达先于IRF9[11]，因而不能排除IRF9在APL中的下调可能是PR下调IRF1的间接结果。

综上所述，我们发现IRF9在APL患者中的表达下调和预后不良相关，并通过构建可诱导表达IRF9的NB4细胞系证实了IRF9促进分化和抑制集落形成的体外抗白血病活性，提示IRF9可作为APL中的潜在靶点产生治疗作用。IRF9在真实世界中的临床预后意义、其体内抗白血病活性及其与RARα融合基因的调控机制仍有待进一步研究。
